# Entorhinal Cortex dysfunction can be rescued by inhibition of microglial RAGE in an Alzheimer’s disease mouse model

**DOI:** 10.1038/srep42370

**Published:** 2017-02-13

**Authors:** Chiara Criscuolo, Veronica Fontebasso, Silvia Middei, Martina Stazi, Martine Ammassari-Teule, Shirley ShiDu Yan, Nicola Origlia

**Affiliations:** 1 Neuroscience Institute, Italian National Research Council, Pisa, 56100 Pisa, Italy; 2 Institute of Cell Biology and Neurobiology, Italian National Research Council, Roma, 00143 Roma, Italy; 3 Santa Lucia Foundation, Roma 00143, Italy; 4 Department of Pharmacology and Toxicology, University of Kansas, Lawrence, KS 66045, USA

## Abstract

The Entorhinal cortex (EC) has been implicated in the early stages of Alzheimer’s disease (AD). In particular, spreading of neuronal dysfunction within the EC-Hippocampal network has been suggested. We have investigated the time course of EC dysfunction in the AD mouse model carrying human mutation of amyloid precursor protein (mhAPP) expressing human Aβ. We found that in mhAPP mice plasticity impairment is first observed in EC superficial layer and further affected with time. A selective impairment of LTP was observed in layer II horizontal connections of EC slices from 2 month old mhAPP mice, whereas at later stage of neurodegeneration (6 month) basal synaptic transmission and LTD were also affected. Accordingly, early synaptic deficit in the mhAPP mice were associated with a selective impairment in EC-dependent associative memory tasks. The introduction of the dominant-negative form of RAGE lacking RAGE signalling targeted to microglia (DNMSR) in mhAPP mice prevented synaptic and behavioural deficit, reducing the activation of stress related kinases (p38MAPK and JNK). Our results support the involvement of the EC in the development and progression of the synaptic and behavioural deficit during amyloid-dependent neurodegeneration and demonstrate that microglial RAGE activation in presence of Aβ-enriched environment contributes to the EC vulnerability.

The entorhinal cortex (EC), an essential component of the medial temporal lobe long-term-memory system, represents the main source of input to the hippocampus and the primary target of hippocampal outputs. The EC inputs to the hippocampus arise primarily from the superficial layers (II and III), while the deep layers (layers V and VI) receive hippocampal projections^1^. The EC can be subdivided in the medial (MEC) and lateral area (LEC) which have distinct functional properties. The MEC superficial layers contain several cell types which are spatially modulated, whereas adjacent neurons in the LEC show only sparse spatial modulation^2^,^3^,^4^,^5^ and respond instead to olfactory stimuli^6^,^7^,^8^ and somatosensory information^9^,^10^,^11^,^12^. More recently, an important role has been ascribed to the EC in object recognition and novelty detection^13^. The EC represents therefore a crucial site for memory formation as it integrates spatial information processed from the MEC neurons with non-spatial information processed from the LEC neurons^14^,^15^,^16^,^17^. The involvement of the EC in cognitive processes is relevant for neurodegenerative disorders such as Alzheimer’s disease (AD), as it is one of the earliest affected brain regions^18^. This might be the consequence of a particular vulnerability of the superficial layer II neurons, that are susceptible to the deleterious consequences of aging and AD^19^, resulting in a significant reduction of their number in the early stages of the disease^20^. In addition, the typical hallmarks of AD, such as the presence of amyloid protein and neurofibrillary tangles, are seen primarily in the EC in mild AD and “spread” to the hippocampus and other cortical areas as the disease progresses^21^. In an AD mouse model, selective overexpression of mutant amyloid precursor protein (APP) predominantly in layer II/III neurons of the EC caused an aberrant excitatory cortico-hippocampal network activity leading to behavioural abnormalities^22^. Thus, the hypothesis has been raised that neurodegeneration primarily observed in EC neurons may cause trans-synaptic deficits initiating the cortical-hippocampal network dysfunction in mouse models and human patients with AD.

Despite these important findings, the functional aspects of the EC superficial layer intrinsic circuitry in AD models have been seldom analyzed. In our previous works, we demonstrated that superficial Layer II horizontal connections are vulnerable to the effects of exogenously applied β-amyloid protein (Aβ) oligomers^23^,^24^,^25^. Here, we characterized the time-course of synaptic impairment of the EC layer II in human amyloid precursor protein J20 transgenic mice (mhAPP), displaying progressive accumulation of human Aβ-peptide. We also investigated whether EC synaptic changes were associated with behavioural abnormalities as assessed by associative memory test that depend on EC functional integrity^26^,^27^. Considering the relevance of Aβ peptide in the pathogenesis of AD, the identification of its cell surface target, as well as the mechanisms of signal transduction, which follow this interaction are important issues. In this regard, it has been speculated that the receptor for advanced glycation end products (RAGE), a multi-ligand receptor of the immunoglobulin superfamily, acts as a binding site on the cell surface for the Aβ protein^28^. It was demonstrated the ability of RAGE in mediating the effects of Aβ on different cell-type, such as neurons, glia and endothelial cells^29^,^30^,^31^,^32^,^33^. In particular, a prominent role for RAGE expressed in microglia emerged as a factor contributing to Aβ-dependent neuronal dysfunction^24^. Indeed, inhibition of microglial RAGE leads to a decrease of the activation of the signal cascade induced by Aβ peptide, involving pro-inflammatory factors^30^,^34^,^35^,^36^ and the activation of protein kinase stress-correlated, such as JNK and p38 MAPK^24^,^37^. We therefore verified the protective effect of selective RAGE inhibition using transgenic mice expressing a dominant-negative form of RAGE targeted to microglia (DNMSR) that were crossed with mice overexpressing APP, obtaining double transgenic mhAPPxDNMSR mice.

We show that EC synaptic function is early affected in mhAPP mice and associated with an impairment in remembering novel object/place and object/place/context associations. More importantly, we demonstrated that inactivation of microglial RAGE in mhAPP mice prevented the activation of p38MAPK and JNK and protected from synaptic and behavioural deficit.

## Results

### EC intrinsic circuitry synaptic function is progressively affected in mhAPP mice

**Figure 1:**
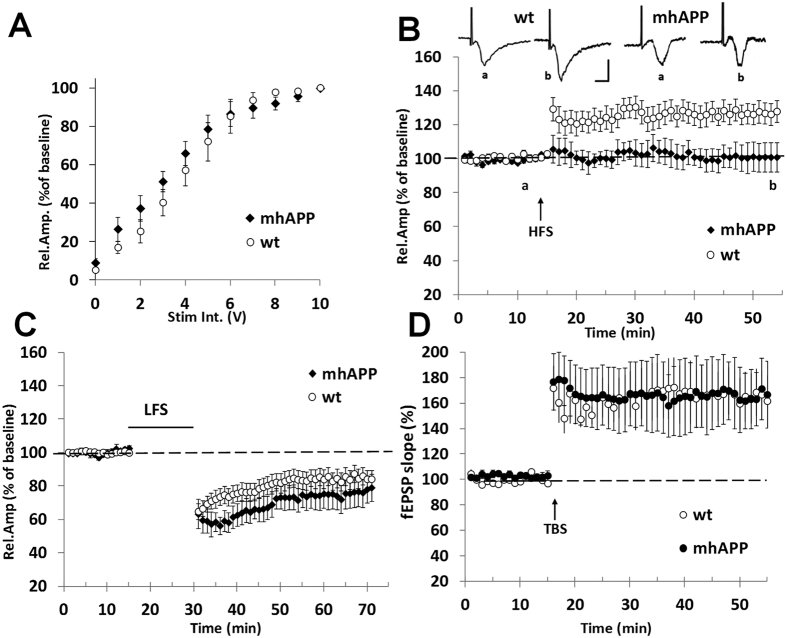
Synaptic plasticity impairment in EC slices from 2 month old mhAPP mice. Field potentials were recorded in EC superficial Layer II after stimulation of the same layer. (**A**) Input– output curves; the relative amplitude (Rel. Amp.) as a function of stimulus intensity (Stim. Int.) measured in volts (V) did not show significant differences between mhAPP (black diamonds) and WT (open circles). (**B**) LTP expression was induced by HFS, applied after 15 min of baseline recording. The LTP was induced by HFS stimulation in WT EC slices (open circles), whereas LTP expression was absent in mhAPP slices (black diamonds); insert show representative field potentials recorded either before (a) or 40 min after (b) HFS in WT and mhAPP EC slices (calibration: 1 mV, 5 ms). (**C**) The LTD expression is not affected in entorhinal cortex slices from 2 month old mhAPP mice as it was reliably inducible by LFS (black diamonds) and comparable to WT (open circles). (**D**) Field potentials were recorded in hippocampal DG after perforant pathway stimulation; LTP expression in the DG induced by TBS did not differ between WT (open circles) and mhAPP (black circles). Error bars indicate SEM.

**Figure 2:**
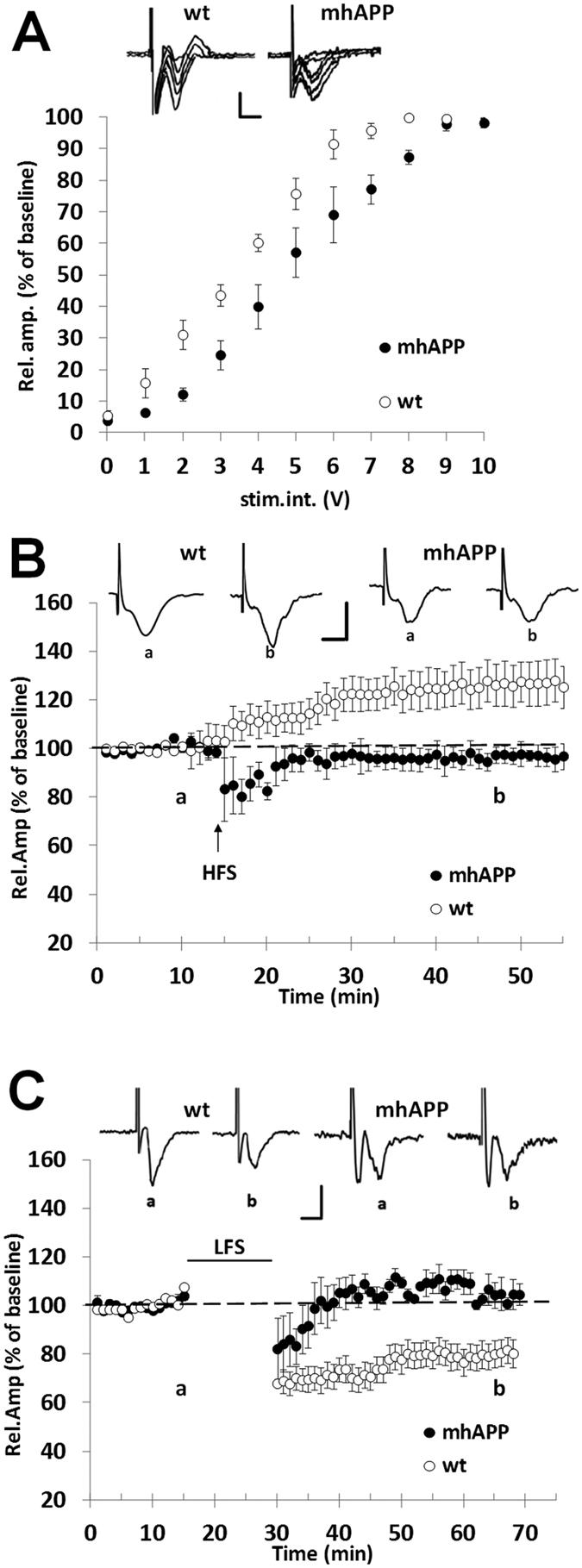
Synaptic plasticity impairment in EC slices from 6 month old mhAPP mice. A significant difference was observed in basic synaptic transmission between WT and mhAPP (**A**) the plot represents the relationship between the amplitude of the response and the stimulus intensity under basal conditions (input–output curve). In (**C**) long term potentiation (LTP) was normally expressed in EC slices from WT mice (open circles). In contrast, LTP magnitude was not inducible by HFS in mhAPP slices (black circles); insert shows representative FPs recorded during baseline (a) or after HFS stimulation (b). Moreover, LFS stimulation (**D**) was not capable of modifying FPs amplitude in EC slices from 6 month old mhAPP mice (black circles), whereas it was capable of inducing the LTD in WT slices (open circles); insert shows representative FPs recorded during baseline (a) or after LFS stimulation (b). In (**C**,**D**) scale bars correspond to 0.5 mV and 5 ms.

Previous evidences have documented the vulnerability of the Entorhinal Cortex to the effects of exogenously applied oligomeric Aβ^24^,^25^. These results prompted us to investigate EC vulnerability in a mouse model characterized by progressive accumulation of human Aβ, such as mice expressing a mutant form of human APP (mhAPP)^38^. First, we investigated synaptic function in 2 month old mhAPP mice and age-matched non transgenic littermate (WT). At this age, mhAPP mice did not show amyloid plaque deposition but a significant increase in Aβ levels, particularly Aβ(1–42), was detectable in the hippocampus compared to wild-type APP transgenic animals^38^. Using an ELISA assay, we confirmed that Aβ(1–40) and (1–42) levels are detectable in protein extract prepared from 2 month old mhAPP EC slices (see Supplementary Fig. S1). Synaptic transmission was evaluated by measuring the amplitude of FPs as a function of stimulus intensity. The input– output curves recorded in slices from mhAPP mice and WT controls did not differ significantly and were clearly overlapping (Fig. 1A; n = 6 slices, 3 mice and n = 8 slices, 4 mice respectively). This suggests that EC synaptic transmission is not altered at an early stage of AD-like phenotype in mhAPP mice. However, HFS of the EC superficial layer could not induce an LTP in mhAPP slices (101 ± 5.5% of baseline, mice n = 4; slices n = 8; *p* = 0.063 *vs*. baseline; Fig. 1B), whereas it elicited a potentiation in slices from age-matched WT mice (128 ± 6% of baseline, mice n = 5; slices n = 10; *p* < 0.001 *vs*. baseline; Fig. 1B). In contrast, LTP can be elicited in EC slices from 1 month old mhAPP mice (data not shown). To verify whether other forms of synaptic plasticity are affected in EC superficial layer of 2 month old mhAPP mice, we investigated the expression of LTD. According to our previous study^24^, LFS stimulation is capable of inducing a stable LTD in WT slices (84 ± 5.1% of baseline, mice n = 4; slices n = 10; *p* < 0.05 *vs*. baseline; Fig. 1C) and a similar long-lasting depression was obtained also in mhAPP slices (79 ± 6.5% of baseline, mice n = 3; slices n = 6; *p* < 0.05 *vs*. baseline; Fig. 1C). Thus, LTP deficiency in the EC represents an early sign of synaptic plasticity impairment in mhAPP mice. A reduction of LTP was previously demonstrated in this mouse model at a later stage (4 months of age) in the Perforant pathway/Dentate gyrus circuitry, which represents the major output projection of the EC^39^. To clarify whether LTP in the DG was altered in 2 month old animals we recorded FPs from granule cell layer after Perforant pathway stimulation. As reported in Fig. 1D, no significant difference was found in LTP expression between WT and mhAPP slices (162 ± 19%, mice n = 5, slices n = 9, and 166 ± 20% of baseline mice n = 3, slices n = 5, respectively). Together, these results demonstrate that LTP impairment is specifically present and primarily observed in the EC of young mhAPP mice. To further investigate the impact of progressive amyloid accumulation on EC synaptic dysfunction we analyzed 6 month old mhAPP mice. At this age diffuse amyloid immunoreactivity was observed in the molecular layer of the dentate gyrus, and in the neocortex^38^. Moreover, measurement by ELISA revealed that Aβ(1–40) and (1–42) levels in the entorhinal cortex were significantly higher in 6 month old than in 2 month old mhAPP slices (Supplementary Fig. S1). In agreement with what reported at 2 months of age LTP impairment was observed in mhAPP slices obtained from 6 month old animals. As shown in Fig. 2B, HFS induced a stable LTP in control WT (126 ± 8% of baseline, mice n 4; slices n = 7; *p* < 0.05 *vs*. baseline) but not in mhAPP EC slices (96.7 ± 3.6% of baseline, mice n = 4; slices n = 6; p > 0.05 *vs*. baseline; *p* < 0.05 *vs*. WT). However, at this stage of neurodegeneration, basal synaptic transmission and LTD were also affected. The input/output curve were significantly different between WT and mhAPP EC slices (at half maximal stimulation mean rel. Amp. were 75.7 ± 5%, mice n = 3, slices n = 6, and 57 ± 7% of baseline, mice n = 3, slices n = 6, respectively; Fig. 2A). In addition, LFS failed to induce a significant change in FPs amplitude in mhAPP slices (104 ± 4% of baseline, mice n = 4; slices n = 8; *p* > 0.05 *vs*. baseline) but was capable of inducing a LTD in age-matched WT control slices (80 ± 5% of baseline, mice n = 4; slices n = 7; *p* < 0.01 *vs*. baseline; *p* < 0.01 *vs*. mhAPP). These data suggest that synaptic function is progressively affected in mhAPP mice and that the first alteration is observed in the EC intrinsic circuitry.

### Inhibition of RAGE signalling in microglia prevents EC synaptic impairment in mhAPP mice

**Figure 3:**
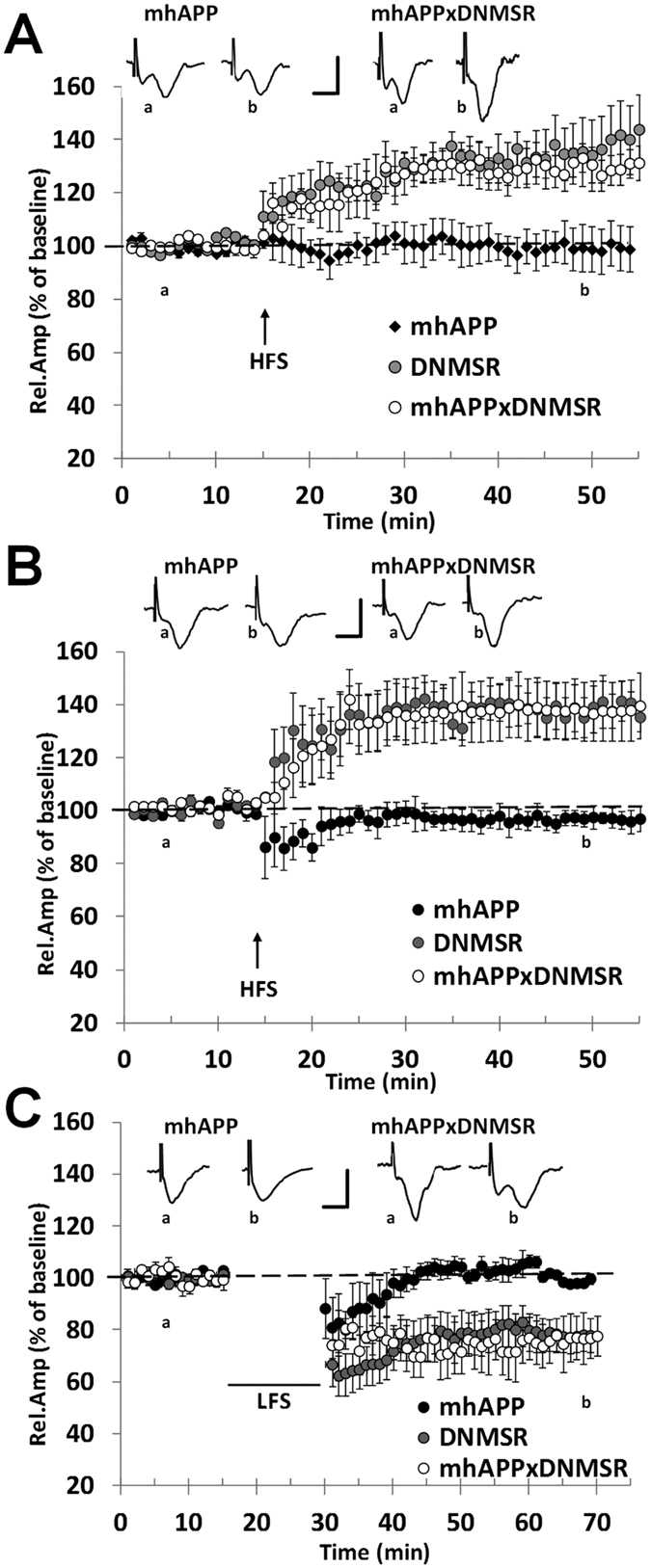
Inhibition of microglial RAGE prevents EC synaptic impairment in mhAPP mice at different stages of neurodegeneration. In 2 month old mice (**A**) deficiency of RAGE did not alter LTP expression in DNMSR EC slices (grey circles) and was sufficient to prevent LTP impairment in double mhAPPxDNMSR transgenic EC slices (open circles), with respect to single mhAPP transgenic slices (black diamonds); insert shows representative FPs recorded during baseline (a) or after HFS stimulation (b). The protective effect provided by RAGE signaling inhibition was confirmed in older animals (6 months of age); in (**B**) the LTP was normally expressed in either DNMSR (grey circles) or mhAPPxDNMSR slices (open circles) with respect to mhAPP slices (black diamonds); insert shows representative FPs recorded during baseline (a) or after HFS stimulation (b). Similarly, LTD was inducible by LFS in EC slices from 6 month old DNMSR (grey circles) and mhAPPxDNMSR (open circles) mice with respect to slices from age-matched mhAPP mice (black diamonds); insert shows representative FPs recorded during baseline (a) or after LFS stimulation (b). In (**A**–**C**) scale bars correspond to 0.5 mV and 5 ms. Error bars indicate SEM.

Activation of RAGE in neurons was involved in synaptic dysfunction induced by exogenous application of Aβ in the EC^24^,^25^; in particular increasing synthetic Aβ concentration up to a micromolar level induces RAGE activation in microglial cells that progressively affects basal synaptic transmission and LTD, in addition to LTP^24^. These results prompted us to verify the hypothesis that inhibition of RAGE signalling in microglia would represent the best strategy to prevent the synaptic effects of Aβ accumulation in mhAPP mice. First, we recorded EC slices prepared from either DNMSR or double transgenic mhAPPxDNMSR 2 month old mice. As previously reported, deficiency of RAGE in microglia does not affect basal synaptic transmission and LTD in EC slices^24^. As reported in Fig. 3A, LTP induction and maintenance were also not affected in DNMSR slices (139 ± 12% of baseline, mice n = 4; slices n = 8; *p* < 0.05 *vs*. baseline) and were comparable to WT controls. Remarkably, deficiency of RAGE in microglia was able to prevent synaptic plasticity impairment induced by mutant APP overexpression. The mean LTP in slices from mhAPPxDNMSR mice was significantly higher respect to slices from single mhAPP transgenic mice (130 ± 5%, mice n = 4, slices n = 6, *vs*. 99 ± 6% of baseline mice n = 3, slices n = 6 respectively, *p* < 0.001; Fig. 3A) and was comparable to that recorded in slices from DNMSR mice (*p* = 0.716). As reported above at a later stage of neurodegeneration, corresponding to 6 months of age, synaptic impairment in mhAPP slices involved basic synaptic transmission and LTD expression. According to what reported in younger animals, no significant differences were found in synaptic transmission between DNMSR and WT slices obtained from 6 month old mice (Supplemental Fig. S2); in addition HFS was capable of inducing a stable LTP in DNMSR slices (138 ± 7% of baseline ampl., mice n = 4, slices n = 6, *p* < 0.001 *vs*. baseline; Fig. 3B). More importantly, at this later stage, deficiency of RAGE in microglia rescued basal synaptic transmission (Supplemental Fig. S2) and LTP expression in double transgenic mhAPPxDNMSR slices compared to single mhAPP slices (137 ± 11%, mice n = 3, slices n = 6, *vs*. 97 ± 3% of baseline mice n = 3, slices n = 6 respectively, *p* < 0.05; Fig. 3B). Moreover, RAGE signalling inhibition protected mhAPP slices from LTD impairment. According to what reported above, LTD was completely abolished in 6 month old mhAPP slices (100 ± 7%, mice n = 3, slices n = 6; *p* = 0.160 *vs*. baseline; Fig. 3C); in contrast, after LFS stimulation a statistically significant LTD was induced in mhAPPxDNMSR slices (76 ± 8%, mice n = 3, slices n = 6; *p* < 0.001 *vs*. baseline; Fig. 3C) that was comparable to that obtained in either DNMSR (78 ± 6%, mice n = 3, slices n = 6; *p* < 0.001 *vs*. baseline; *p* = 0.359 *vs*. mhAPPxDNMSR; Fig. 3C) or WT controls slices (80 ± 5% of baseline, mice n = 4; slices n = 7; Fig. 2C; *p* = 0.294 *vs*. mhAPPxDNMSR). Therefore, microglial RAGE activation in presence of APP overexpression is relevant to induce progressive synaptic alteration in the EC superficial Layer II.

### Entorhinal cortex dependent behaviour is early affected in mhAPP but not in mhAPPxDNMSR mice

**Figure 4:**
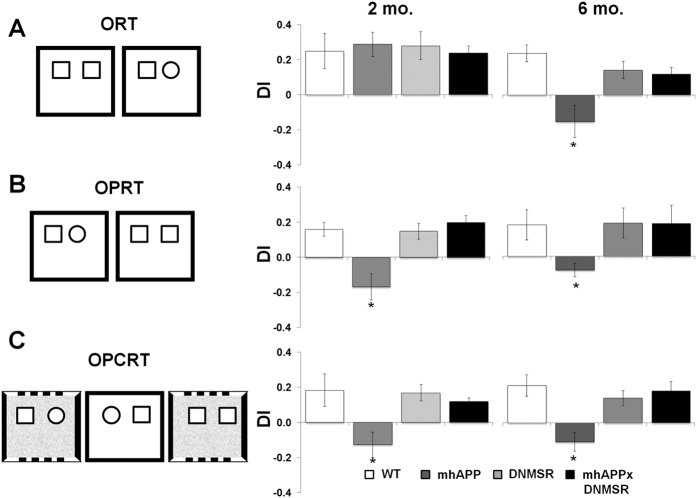
Behavioural analysis. In (**A**–**C**) left panels report a schematic depiction of the sample and test trials within the novel object recognition tasks (ORT), novel object place recognition task (OPRT) and novel object place/contest recognition task (OPCRT), respectively; In (**A**–**C**) right panels, plots represent the average discrimination indices (DI) calculated for each group of either 2 month old or 6 month old mice. Data are presented as mean ± SEM; *p < 0.05 *vs*. other groups.

The above data demonstrate that RAGE expressed in microglial cells is an important co-factor that participates in EC synaptic dysfunction in mhAPP mice. Therefore, the next step was to investigate whether synaptic dysfunction was associated with impairment in EC-dependent memory and to identify the specific role of RAGE in these events by its selective inhibition in microglia. The Lateral EC (LEC) is required for the elaboration of non-spatial information involved in the formation of episodic memories in rodents^13^ and lesions of the LEC cause a selective impairment in memory tasks requiring the combined elaboration of spatial (referred to context and objects position) and non spatial (referred to objects) information^27^. First, we confirmed that WT mice with selective lesions of the LEC show an impairment in the execution of the OPRT and OPCRT, which both depend on elaboration of spatial and non spatial details (see Supplemental Fig. S3)^27^. This data indicates that the integrity of LEC is required for complex memories. We therefore analysed memory performance of 2 months old male mhAPP mice and WT littermate in the OPRT and OPCRT tasks, and compared the performances of mice in the less cognitively-demanding ORT to investigate how synaptic dysfunction in the EC of young mhAPP mice impacts on cognition. As reported in Fig. 4A, the average discrimination indices (DI) in the ORT were not significantly different between WT and mhAPP mice (0.25 ± 0.1, n = 6; *vs*. 0.29 ± 0.7; *p* = 0.99). Mice from both genotypes had DI significantly greater than chance demonstrating that they preferred exploring the novel object rather than the old. In contrast, the performance of mhAPP mice in the OPRT and OPCRT revealed an impairment in the ability to discriminate the novel object in relation to both its position and the surrounding context. The average DI for mhAPP mice were not significantly different respect to what expected by chance either for OPRT (0.25 ± 0.1, n = 6; *p* = 0.074; Fig. 4B) or OPCRT (−0.13 ± 0.05, n = 6; *p* = 0.132, Fig. 4C) and were significantly different from DI of age-matched WT mice (*p* < 0.05 *vs*. 0.16 ± 0.09, n = 6; and 0.18 ± 0.09, n = 6; for OPRT and OPCRT respectively). According to what described for electrophysiological findings, the inhibition of RAGE in microglia was sufficient to prevent behavioural impairment in mhAPP mice, as mhAPPxDNMSR mice showed a preference toward novelty not only in the simplest ORT but also in the more complex OPRT and OPCRT versions of the task. The DI of mhAPPxDNMSR mice calculated after the OPRT and OPCRT were significantly greater than chance (0.20 ± 0.04, n = 6; *p* = 0.004, Fig. 4B; and 0.12 ± 0.02, n = 6; *p* = 0.002, Fig. 4C) and significantly different from those of mhAPP mice (*p* < 0.05, Fig. 4B and C); while they were comparable to those found in WT and DNMSR mice. It has to be noticed that there was no significant difference in the total amount of time spent in exploring objects (time spent at novel + familiar objects) between the different groups. Moreover, no significant differences were observed between groups in the total locomotor activity and exploration (time spent in the centre *vs*. periphery of the box), during the habituation phase (Supplemental Fig. S4). These data suggest that a first decline in complex memories can be observed in young mhAPP mice that can be prevented in double mhAPPxDNMSR mice. At a later stage of neurodegeneration (6 months of age), mhAPP mice confirmed the impairment in performing the associative tasks, as the average DI in the OPRT (−0.07 ± 0.04, n = 6; *p* = 0.099; Fig. 4B) and OPCRT (−0.11 ± 0.05, n = 6; *p* = 0.098, Fig. 4C) were not significantly different from what expected by chance and were significantly different from DI calculated for age-matched WT mice (*p* < 0.05 *vs*. 0.18 ± 0.06, n = 6; and 0.21 ± 0.06, n = 6; for OPRT and OPCRT, respectively). In contrast to what reported above in younger animals, 6 month old mhAPP mice also displayed a significant deficit in remembering the familiar object in the simplest task (mean DI for ORT was −0.14 ± 0.09, n = 6; *p* = 0.163; Fig. 4A) with respect to age-matched WT (mean DI was 0.24 ± 0.05, n = 6; *p* = 0.004). However, selective inhibition of RAGE confirmed its protective effect and completely prevented the behavioural deficits in 6 month old mhAPP mice. Indeed, at this age, mhAPPxDNMSR mice preferred the novel object in the ORT (mean DI was 0.12 ± 0.03, n = 6; *p* = 0.010) in a comparable manner respect to age-matched WT (*p* = 0.510; Fig. 3A) and DNMSR mice (*p = *0.049; Fig. 3A). Moreover, mhAPPxDNMSR mice showed a preference for novel associations in the OPRT (mean DI was 0.19 ± 0.07, n = 6; *p* = 0.048; Fig. 4B) and OPCRT (mean DI was 0.18 ± 0.05, n = 6; *p* = 0.015; Fig. 4C) and DI were not significantly different from those observed in age-matched WT and DNMSR mice (Fig. 4B and C). No significant differences were found between the groups of 6 month old mice in the total time spent in exploring object and in the locomotor activity; however either mhAPP or mhAPPxDNMSR mice spent a greater amount of time in exploring the periphery of the box during the habituation phase with respect to WT and DNMSR mice (Supplemental Fig. S4), this suggests that deficiency of RAGE ameliorate memory in older mhAPP mice without any effect on their increased anxiety.

### Altered dendritic spine morphology in mhAPP mice is rescued by RAGE inhibition in microglia

**Figure 5:**
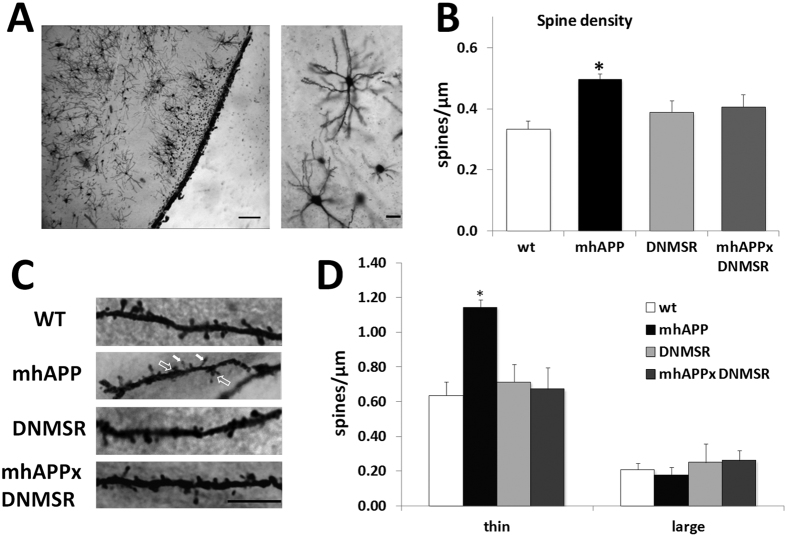
Analysis of spine density and morphology of LEC neurons in 2 month old mice. (**A**) Representative Golgi-stained EC slices and layer II neurons (scale bar = 250 μm). Magnification (40×, scale bar = 100 μm) shows a pyramidal (bottom) and a multiform (upper) neuron in the lateral EC. (**B**) Plots reporting the average spine density calculated for each genotype. (**C**) Representative dendritic segments of Golgi-stained EC layer II neurons are reported for each genotype (100× magnification, scale bar = 5 μm). Spines were classified as thin or large based on their morphology (shape and dimension); examples of thin (white arrows) and large (empty arrows) spines are reported for mhAPP mice. In (**D**) The plots represent the average density of thin and large spines calculated for ach genotype. Data are presented as mean ± SEM (*p < 0.05).

Our data demonstrate that early synaptic changes occurring in mhAPP mice are associated with behavioural deficits. As previously reported, Aβ peptide exerts a regulatory control over excitatory synaptic function, which can be either a positive or a negative regulation depending on peptide concentration^40^. We therefore wanted to complete our investigation on EC neuronal plasticity by analysing the effect of mutant APP over-expression on dendritic spines, the locus of excitatory synapses. To this aim, two month old mhAPP mice and littermates WT controls were sacrificed and their brains processed for Golgi-Cox staining. The number and morphology of dendritic spines were assessed along pyramidal and multiform neurons with cell bodies lying in Layer II of the lateral EC (Fig. 5A). Data in Fig. 5 indicate that neurons from mhAPP mice showed a significant increase in spines density as compared to wild type controls (0.50 ± 0.01 *vs*. 0.33 ± 0.03 spines/μm; p < 0.05; n = 6 mice, Fig. 5B), and that this increase depends on a different distribution of thin rather than large spines (Fig. 5D). A significantly greater proportion of thin spines was indeed observed in mhAPP mice with respect to wild type (1.14 ± 0.04, n = 5 *vs*. 0.63 ± 0.08 spines/μm, n = 5 mice; p < 0.05; Fig. 5D), while the proportion of mushroom spines did not significantly differ between mhAPP and WT mice (0.18 ± 0.05, n = 5 *vs*. 0.21 ± 0.03 spines/μm, n = 5 mice; in mhAPP and WT respectively; *p* > 0.05 in Fig. 5D). We next asked whether RAGE was involved in mhAPP-associated changes in dendritic spines. To answer this question, we measured dendritic spines in brain slices collected from mhAPPxDNMSR mice. Results reported in Fig. 5B–D show that the number of dendritic spines was unvaried in DNMSR mice as compared to wild type controls (0.39 ± 0.03 spines/μm, n = 6 mice; p = 0.565 *vs*. WT; Fig. 5D), moreover the total number of spines in mhAPPxDNMSR mice was significantly different from that of single mhAPP (0.41 ± 0.03 spines/μm, n = 6 mice, p < 0.05; Fig. 5D) and was comparable to either DNMSR (*p* = 0.548) or WT mice (*p* = 0.35). The rescue of synaptic density in mhAPPxDNMSR mice was associated with a return to control value of thin immature spines (0.67 ± 0.12, n = 6 mice; p < 0.05 *vs*. mhAPP; Fig. 5D).

Altogether the above data indicate that an amyloid enriched environment lead to the aberrant production of thin and possibly dysfunctional spines in the EC of mhAPP mice and that inhibition of RAGE in microglial cells was sufficient to prevent such amyloid-associated increase of dendritic spines.

### The kinases p38MAPK and JNK are differently activated in the EC of mhAPP mice at different stages of neurodegeneration

**Figure 6:**
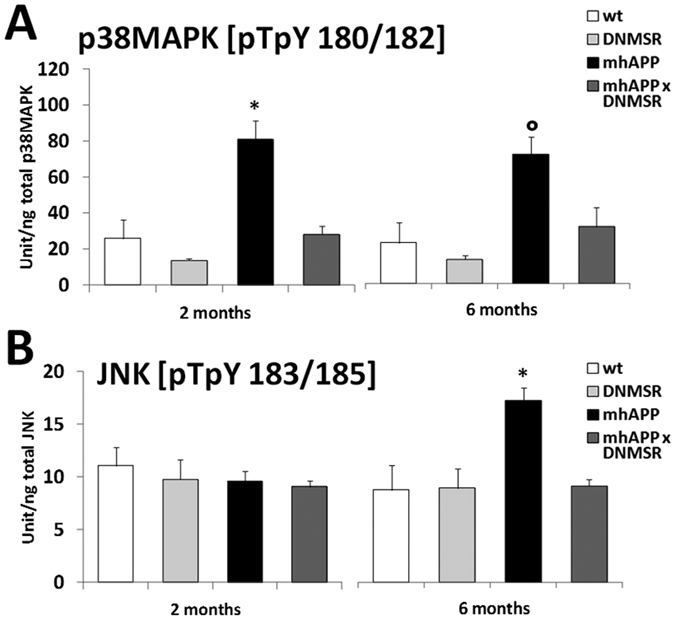
The increase of p38 MAPK/JNK phosphorylation in the EC of mhAPP mice is prevented by microglial RAGE inhibition. In (**A**) the plot represents averaged phospho-p38 MAPK levels measured using ELISA and expressed as unit/total content of p38 MAPK protein. Tissue levels of phospho-p38 MAPK were significantly higher in the EC of mhAPP mice with respect to control WT slices (*°*p* < 0.05) both in 2 and in 6 month old animals. Selective deficiency of RAGE signalling in microglia (DNMSR) did not modify basal level of phospho-p38 MAPK and was capable of preventing the increase of phospho-p38 MAPK in mhAPPxDNMSR mice at either 2 or 6 months of age. In (**B**) the plot represents averaged phospho-JNK levels measured using ELISA and expressed as unit/total content of JNK protein. Phospho-JNK levels were increased in the EC of 6 month old mhAPP mice (*p < 0.05 *vs*. WT) but not in that of 2 month old mhAPP mice. Deficiency of RAGE signalling in microglia (DNMSR) did not modify basal level of phospho-JNK but was able to prevent the increase of phospho-JNK in mhAPPxDNMSR mice.

**Figure 7:**
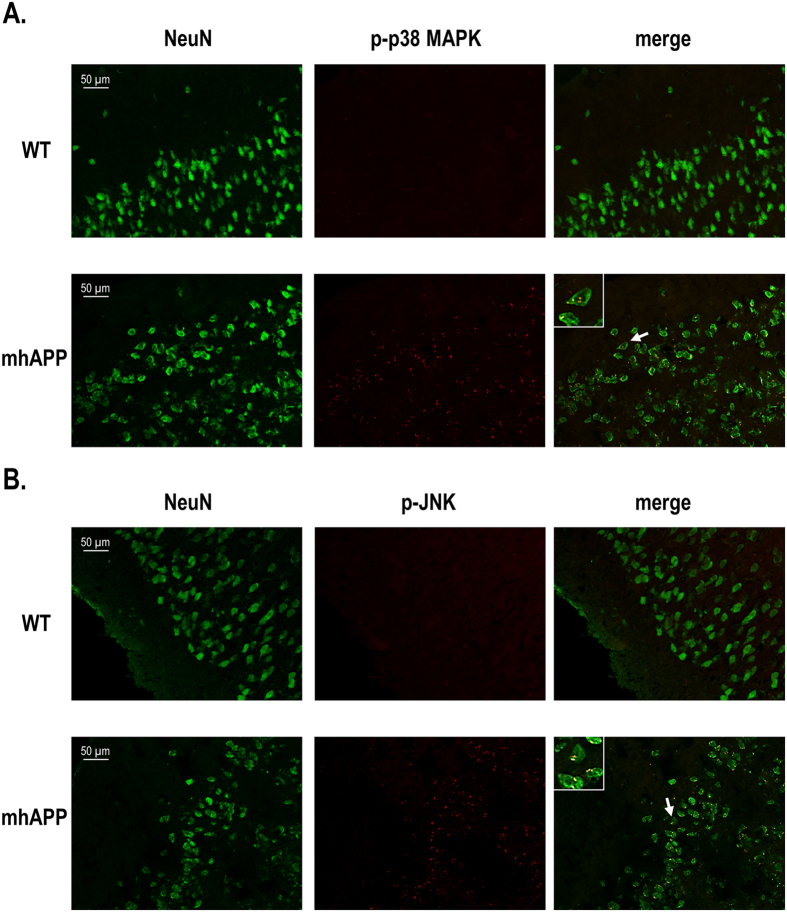
Immunolocalization of phosphorylated p38 MAPK/JNK in the EC of mhAPP mice. (**A**) NeuN (neuronal) labeled cells (left column) and phosphorylated-p38 fluorescent staining (p-p38, middle columns). Representative images (20×) show that p-p38 fluorescent signal was increased in EC superficial layers of 2 month old mhAPP mice (middle raw) respect to age-matched wild-type (upper panels). Phospho-p38 immunofluorescence co-localizes with the neuronal marker NeuN (merge in right column and enlarged neuron indicated by the arrow in the insert). (**B**) NeuN (neuronalmarker) labeled cells (left column) and phosphorylated-JNK (p-JNK) immunoreactivity (middle columns). Cells in the right column (merge) are double-labeled forNeuN and p-JNK. Phosphorylated form of JNK was rarely observed in 6 month old WT mice (upper raw). Representative images (20×) show that p-JNK immunofluorescence is increased in the layer II/III of mhAPP EC (middle raw) respect to age-matched WT (upper raw). The pattern of p-JNK immunolabeling strongly co-localize with the neuronal marker NeuN (merge in right column and insert of a neuron indicated by arrow at higher magnification). Scale bar = 50 μm.

To gain further insight in to the signalling cascade that might be modulated by microglial RAGE in mhAPP mice, we investigated the role of p38MAPK and JNK. We focused our attention to these kinases because they were previously shown to be strongly activated in cultured neurons and EC slices exposed to high levels of Aβ^24^,^25^,^41^. To better clarify whether the level of activation of stress-related kinases changes depending on the stage of neurodegeneration and by the cell-specific RAGE activation, we measured tissue levels of phosphorylated p38MAPK and phosphorylated JNK in EC slices from either 2 or 6 month old mice. As reported in Fig. 6A, mhAPP slices that were collected from 2 month old mice showed a significant increase in phospho-p38MAPK tissue levels with respect to age-matched WT controls (81.38 ± 28.7 U/ng, n = 6 slices, 4 mice *vs* 26.04 ± 10.2 U/ng, n = 7 slices, 4 mice; *p* < 0.001; Fig. 6A). Activated p38MAPK was particularly evident in EC layer II/III of 2 month old mhAPP mice, compared to the immunoreactivity found in age-matched WT mice (Fig. 7A). Co-localization of p-p38 MAPK with the neuronal marker NeuN was observed either in the mhAPP or WT slices (Fig. 7A). According to what previously reported^24^, selective deficiency of RAGE signaling in microglia (DNMSR) did not modify the level of phospho-p38MAPK in EC slices (13.6 ± 1.4 U/ng, n = 4 slices, 3 mice; *p* = 0.575 *vs* WT; Fig. 6A). However, slices from 2 month old mhAPPxDNMSR mice displayed complete suppression of phospho-p38MAPK with respect to single mhAPP transgenic mice (28.1 ± 4.5 U/ng, n = 6 slices, 4 mice; *p* < 0.001 *vs*. mhAPP; p > 0.05 *vs*. WT and DNMSR; Fig. 6A). A similar increase in phospho-p38MAPK levels was also observed in older mhAPP mice (6 months of age, Fig. 6A) respect to age-matched WT controls (72.9 ± 10.2 U/ng, n = 6 slices, 4 mice *vs* 23.1 ± 11.6 U/ng, n = 7 slices, 4 mice; *p* = 0.010; Fig. 6A). Like in younger animals, selective deficiency of RAGE was capable of preventing the phosphorylation of p38MAPK in mhAPPxDNMSR slices from 6 month old mice (32.09 ± 10 U/ng, n = 6 slices, 4 mice; *p* = 0.041 *vs* mhAPP; p > 0.05 *vs* WT and DNMSR; Fig. 6A). These results confirm that p38MAPK is activated in the EC of mhAPP mice at an early stage and this phenomenon can be modulated by RAGE expressed in microglia.

Unlike p38MAPK, the phosphorylation level of the other kinase JNK was not significantly increased in EC slices from 2 month old mhAPP mice with respect to age-matched WT (9.5 ± 0.9 U/ng, n = 6 slices, 4 mice *vs* 11.1 ± 1.7 U/ng, n = 8 slices, 4 mice; *p* = 0.742; Fig. 6B). A different result was obtained in 6 month old slices. Phosphorylation of JNK was significantly increased in mhAPP slices respect to age-matched WT (17.1 ± 3 U/ng, n = 6 slices, 4 mice *vs* 11.1 ± 1.7 U/ng, n = 6 slices, 4 mice; *p* = 0.008; Fig. 6B) and DNMSR (8.8 ± 1.8 U/ng, n = 4 slices, 3 mice; *p* = 0.014; Fig. 6B). Accordingly, immunolabeling of phospho-JNK was more evident in the EC layer II of 6 month old mhAPP mice respect to age-matched WT controls (Fig. 7B). Indeed, p-JNK immunostaining co-localized with neuronal marker NeuN (Fig. 7B), similarly to what observed for p-p38MAPK. The genetic inhibition of RAGE in microglia was able to maintain phospho-JNK to basal levels in mhAPPxDNMSR slices (9.0 ± 1.4 U/ng, n = 6 slices, 4 mice; *p* = 0.009 *vs* mhAPP; p > 0.05 *vs* WT and DNMSR; Fig. 6B). Therefore, our data revealed a different time-course in the activation of neuronal p38MAPK and JNK in the EC of mhAPP mice, that can be modulated by targeting microglial RAGE.

## Discussion

The present study focuses on the Entorhinal cortex (EC) as a crucial site for the development of amyloid-dependent neurodegeneration. The alterations of EC superficial layer can directly contribute to downstream changes in its primary afferent regions, such as the hippocampus, resulting in aberrant network activity that has been reported in mouse models and human patients with AD^42^,^43^. Previous evidences, including those from our group, have shown a progressive impairment of synaptic function with increasing extracellular Aβ. In particular, synthetic oligomeric Aβ(1–42) in the nanomolar range was shown to specifically inhibit LTP in the hippocampus^44^,^45^,^46^,^47^,^48^,^49^ and cortical areas, including the EC^23^,^37^,^50^; whereas higher micromolar concentrations caused synaptic depression and LTD impairment^24^,^51^,^52^,^53^. Thus, EC intracortical circuitry is vulnerable to the effects of relatively low concentration of Aβ. However, it is difficult to compare the effects of an exogenous application of Aβ oligomers with the endogenous progressive accumulation occurring in the AD brain. Here, we characterized the EC synaptic dysfunction in a mouse model overexpressing mutant human APP. Our results suggest a precise temporal profile and an exact order of involvement of different circuitries during the progression of synaptic dysfunction in mhAPP mice, possibly corresponding to different stages of Aβ accumulation. Although this animal model of AD displays diffuse amyloid accumulation in the brain, LTP disruption is first observed in the intrinsic circuitry of the EC suggesting that it is more vulnerable respect to other area (i.e. hippocampus). The LTP impairment is the first sign of synaptic dysfunction because it is detectable before deficits in basal synaptic transmission and LTD occur. Consistent with LTP deficit, we also reported a significant increase in the number of thin dendritic spines in layer II of Lateral EC. This effect might be indicative of a massive spines formation in absence of maturation of post-synaptic components^54^,^55^. More importantly, we demonstrated that these early synaptic changes were associated with a behavioural impairment of associative memory. A growing body of evidence has linked the LEC to the formation of episodic memory. This can be achieved in the hippocampus by the complex integration of spatial information from the MEC with non-spatial information coming from the LEC^15^,^17^. In particular, a population of LEC cells have been identified, some of them firing at the objects and other cells firing at places where objects had been located on previous trials^13^. Moreover, LEC is required for recognition of objects that have been experienced in a specific context^26^ and the specific lesion of the LEC causes an impairment in the ability to discriminate either novel object/place or novel object/place/context associations without affecting the recognition of a novel object^27^. In agreement with the electrophysiological results, our behavioural analysis of 2 month old mhAPP mice revealed a selective impairment in EC-dependent tasks as associative memories (OPRT and OPCRT) but not non-associative (ORT) memories were affected. With the progression of neurodegeneration, mhAPP mice also display a deficit in hippocampal synaptic plasticity^22^,^39^,^56^ and we reported a consistent reduction in the ability to discriminate the novel object, as revealed by ORT in 6 month old mice. In aggregate, these findings demonstrate the relative contribution of the EC and hippocampus to synaptic and behavioural deficits that varies depending on the stage of amyloid neurodegeneration. The evidence that Aβ accumulation is linked to the activation of inflammatory pathways^57^ raises the key question of whether brain neuroinflammation is involved in the early synaptic and behavioural deficits induced by Aβ load. In particular microglial cells have been repeatedly demonstrated to be involved in the development of AD. This is not only because of the well-known phagocytic activity against amyloid plaques. In fact, Aβ can interact with microglia inducing morphological changes, cell proliferation/migration, and production of an array of pro-inflammatory factors that are capable of interfering with synaptic function^58^. For example, LTP blockade in hippocampus and behavioural deficits induced by either an inflammatory stimulus or Aβ were prevented by pharmacological inhibition of microglia^49^,^59^. Furthermore a neuroprotective effect can be achieved by inhibition of a specific microglial receptor, such as the Chemokine fractalkine receptor (CX3CR1)^60^ and the Complement receptor 3 (CR3)^61^. Similarly, RAGE is a microglial receptor that is able to interact with either Aβ oligomers or aggregate forms of Aβ with higher efficacy respect to Aβ monomers^28^,^62^. Since RAGE is not only expressed in microglia but also in neurons and other non-neuronal cells^30^,^31^,^33^,^63^, it is important to know the role of its cell specific activation under Aβ load. A first evidence that RAGE can contribute to neurodegeneration in a amyloid-rich environment was reported in Arancio *et al*.^64^. In this work the neuronal overexpression of RAGE in the mhAPP mouse model, accelerated the development of synaptic plasticity impairment in the hippocampus and the onset of behavioural deficit. However, further investigation demonstrated the prominent role of RAGE expressed in microglia. Specifically in the EC, synaptic impairment induced by Aβ oligomers can be prevented by selective suppression of RAGE signalling in microglial cells^24^. In line with these observations, targeting microglial RAGE in mhAPP mice was able to prevent neuronal dysfunction in a wide time window; i.e. at a very early stage when synaptic and behavioural deficits are restricted to the EC and at a later stage that involve hippocampal dysfunction. Although we did not restrict the expression of deficient RAGE to the EC, our data suggest that inhibition of Aβ/RAGE interaction would limit the progression of synaptic dysfunction trough a specific pattern of connections. In this regard, it has been recently demonstrated that microvesicles (MVs) released extracellularly by reactive microglia may contribute to the spreading of AD degeneration^65^. In particular, microglial derived MVs are capable of carrying Aβ and promoting its synaptotoxicity moving from neuron-to-neuron through their projecting axons^65^,^66^. Therefore, MVs may represent a novel intriguing mechanism by which activated microglia participate in the trans-synaptic progression of AD degeneration.

Microglial cells can affect synaptic function with multiple mechanisms, including modulation of synaptic pruning either under physiological or pathological conditions^67^. In a recent paper Hong *et al*.^68^,^69^ have demonstrated that complement-dependent pathway and activated microglia are responsible for synapses loss, as revealed by quantification of co-localized pre- and postsynaptic puncta in the hippocampus of 3–4 month old mhAPP mice. However, synapse levels were not altered in 1 month mhAPP brains^68^. In the same AD model the overall density of large mature spines of hippocampal neurons did not change significantly at 4 months of age with respect to non-transgenic animals^56^. As mentioned above, we found a significant increase in the density of thin immature spines in Layer II of lateral EC of mhAPP mice as compared to wild type controls. This aberrant effect was completely prevented in double mhAPPxDNMSR mice. Although this data is only correlative, it suggests that RAGE signalling triggered by the presence of Aβ can contribute to alterations in pruning processes in EC neurons. Other evidences showing that RAGE-dependent inflammatory signalling can modulate neurite outgrowth and dendritic morphology^70^,^71^ support this possibility. However, we cannot exclude that other factors related to the overexpression of APP such as the soluble APPα fragment that would favour spines growth^72^,^73^, can also participate in aberrant spine density in the EC of these mice.

To confirm that microglial RAGE activates specific intracellular signalling cascade(s) in the EC of mhAPP mice, we investigated the phosphorylation levels of p38MAPK and JNK. We focused our attention to these kinases as they are strongly activated in EC slices exposed to high levels of Aβ^24^,^25^,^41^. Furthermore, the stimulation of RAGE in microglialcells leads to the co-activation of JNK and p38MAPK; these kinases in turn are capable of modulating the release of pro-inflammatory mediators, such as IL-1β a molecule that can affect synaptic function^37^,^74^,^75^,^76^. We have identified a different profile of kinases activation in the EC of mhAPP mice. In particular, p38MAPK but not JNK phosphorylation was increased in the EC of 2 month old mhAPP mice, whereas both kinases were significantly activated in 6 month old animals. In addition we found that these changes in kinases activation are present in EC layer II neurons as revealed by their co-localization with the neuronal marker NeuN. This is in agreement with previous findings showing that acute supply of a lower Aβ concentration activates neuronal p38MAPK leading to LTP inhibition, while JNK phosphorylation was involved in the effect induced by higher Aβ concentration causing LTD blockade and altered glutamatergic synaptic transmission^24^,^25^,^51^,^52^,^53^. Even though it is difficult to correlate EC biochemical/synaptic changes to the hippocampal dependent behavioural (ORT) impairment in 6- month old AD animals, we might speculate that activation of stress signalling pathways in the EC (p38 and JNK) contribute to the exacerbation of EC dysfunction and trans-synaptic progression of neurodegeneration in target area. Deficiency of RAGE signalling prevented p38MAPK and JNK phosphorylation in the EC of mhAPP mice either at the early or the late stage. These data suggest that RAGE dependent activation of p38MAPK in the EC of mhAPP mice is a an event associated to early synaptic plasticity and behavioural deficits. This might have important implications since p38MAPK phosphorylation is elevated in the AD brain^77^ and isoform selective p38αMAPK inhibitors have been developed that attenuates disease progression in AD models^78^.

In summary activation of a RAGE inflammatory signalling *in vivo* caused by an Aβ enriched-environment represents an important early event during the progressive EC dysfunction. Microglial RAGE interaction with Aβ may therefore contribute to trigger cognitive dysfunction in vulnerable brain areas resulting in the spreading of AD pathology.

## Materials and Methods

### Animals

Transgenic mice with signal-transduction-deficient mutants of RAGE in which the cytosolic domain of the receptor has been deleted, thereby imparting a dominant-negative RAGE effect, targeted to microglia (DNMSR, driven by the macrophage scavenger receptor promoter), were originally provided by Dr. S. S. Yan (University of Kansas, Lawrence, Kansas). These transgenic animals have been used previously by our group^24^,^35^,^37^. Furthermore, mhAPP-transgenic mice overexpressing an alternatively spliced human APP (hAPP) minigene that encodes hAPP695, hAPP751, and hAPP770 bearing mutations linked to familial AD (V717F, K670N/M671L) have been used (APPsweInd, line J20)^38^. Double-transgenic mice expressing DN-RAGE in microglia and mhAPP were obtained by crossing DNMSR mice to mhAPP mice as described previously^35^. Transgenic mice and their littermate controls (C57BL/6 J background) were used for the *in vitro* electrophysiology and behavioral testing. C57BL/6 J mice (Charles River, Italy) were also used for surgical lesion. All experiments were conducted according to Ministry of Health (the regulatory authority for controlling the use of laboratory animals and ethics on animal experiments in Italy) guidelines (Legislative Decree n. 116/92) and in accordance with the European Community guidelines European Directive 86/609/EEC). The experimental protocol (IACUC document) was approved by the Ministry of Health (n. 192/2000-A).

### EC slices preparation and Electrophysiology

Transgenic mice and their littermate controls were deeply anesthetized using urethane (Sigma, 20% solution, 0.1 ml/100 g b.w., i.p. injections) via intraperitoneal injection and then decapitated after disappearance of the tail pinch reflex. The brain was rapidly removed and thick horizontal sections (400 μm) containing the entorhinal area were made on a vibratome (Leica VT1200S). All above steps were performed in ice cold artificial CSF (ACSF) oxygenated solution (mM: NaCl, 119; KCl, 2.5; CaCl_2_, 2; MgSO_4_, 1.2; NaH_2_PO_4_, 1; NaHCO_3_, 6.2; glucose, 10; HEPES, 10). Before recording, slices were stored for at least 1 h in a recovery chamber containing oxygenated ACSF at room temperature. During electrophysiological recordings, slices were perfused at a rate of 2 ml/min with oxygenated ACSF at 33 ± 1 °C.

Extracellular field potentials (FPs) were recorded as previously described^25^. Basal recording was carried out using stimulus intensity capable of evoking a response whose amplitude was 50–60% of the maximal amplitude. After 15 min of stable baseline, LTP and LTD were induced by high frequency stimulation (HFS, three trains of 100 pulses at 100 Hz, 10 s interval) and low-frequency stimulation (LFS, 900 paired pulses at 1 Hz, the interval between paired pulses was 30 ms) respectively. After HFS and LFS, FPs continued to be monitored every 20 s for at least 40 min. The magnitude of LTP and LTD were calculated as the average of the relative amplitudes (compared to baseline) of FPs recorded in the last 10 min. Values were expressed as mean ± SEM percentage change relative to their mean baseline amplitude. Data collection and analysis were performed in blind by two different operators.

### Behavioural testing for novel object (ORT), novel object-place (OPRT) and novel object-place-context (OPCRT) recognition task

The behavioural testing was carried out within a 60 cm square box with 40 cm high walls. Following 1 week of extensive handling to habituate the mice to the experimenter, mice were individually habituated to the “white” context (3 days) for one hour each day. Behavioural testing proceeded in sequential stages during two consecutive days as follows:

#### ORT

After 5 minutes of exploration in the “white” context, mice were given a sample trial in which they were allowed to explore two copies of an object for 3 minutes. Easily cleanable 3D household objects made from plastic, metal or glass were used. These were approximately the same size as a mouse (in at least one dimension). Mice were then removed from the box and placed in a holding cage for 1 minute inter trial interval (i.t.i.) while box was cleaned and configured for the following trial. In test trial, mice were exposed to a new copy of the object presented in the sample trial (familiar object, FO) and a novel object (NO) and left free to explore the objects for 3 minutes.

#### OPRT

The first two sessions were repeated as above with the only difference that mice were exposed to two different objects during sample trial. In the test trial, mice were exposed to two copies of one of the previously presented object, which were positioned in the same location (familiar OP association) and in the location that previously had held a different object (novel OP association). Therefore, as compared to ORT task, the OPRT requires the codification of both objects and their position.

#### OPCRT

This task, which is more cognitively demanding for mice as compared to ORT and OPRT tasks, was run on day two. This task requires sequential presentation of objects in two different contexts, here named context 1 and 2. Mice were initially exposed to context 1 for 5 minutes of free exploration of two different novel objects (sample trial 1); then, after a short i.t.i., mice were introduced in context 2 (sample trial 2) and exposed to two different copies of the previously presented objects but at opposite positions with respect to sample trial 1. The test phase was run in context 1 and mice were exposed to two further copies of one of the same objects as in sample trial 2. Thus, in the test phase one of the objects had been presented in that location and context before but not in that location within that particular context (novel OPC association), unlike the other object copy, which had been seen in that location within that context before (familiar OPC association). In all the above experiments, mice were judged to be exploring an object when it was in its close vicinity with the nose directed towards it. Exploration time was not counted in moments when the mouse’s nose was directed away from the object even if the mouse was immediately beside or even on top of it. To check for reliability the same separate observer re-scored a subset of videos in a blind fashion for each task and these scores were found to be consistently within 10% of the experimenter’s. For each task we converted observation scores into discrimination indices (discrimination index = (time at novel − time at familiar)/(time at novel + time at familiar)) to determine the rates that mice explored novel versus familiar objects/places/associations. For all the experiments, the objects used and the side for the novel object in the test phase were counterbalanced as much as possible among experimental groups. A schematic drawing of trials for the different tasks is reported in Fig. 4.

### Golgi Cox staining

Animals (2 month old) were deeply anaesthetized with chloral hydrate (500 mg/kg i.p.) and perfused transcardially with 0.9% saline solution. Brains were dissected and immediately immersed in a Golgi-Cox solution (1% potassium dichromate, 1% mercuric chloride, 0.8% potassium chromate) at room temperature for 6 days. At the end of impregnation period, brains were transferred in a 30% sucrose solution at most for 2 days and then sectioned with a vibratome. Coronal brain sections containing EC (100 μm) were collected and mounted on gelatinized slides, stained according to the method described by Gibb and Kolb^79^, and covered with permount.

### Spine Density and morphology measurement

Spine density and shape analyses were performed on brains slices of transgenic mice and their littermate controls mice after Golgi-Cox impregnation method. Fully impregnated pyramidal neurons of the lateral part of the EC were identified under low magnification (20 ×/0.5 NA). In each brain, 11 neurons displaying dendritic tree without obvious truncations and balanced per hemisphere were analyzed under higher magnification (63 ×/0.75 NA). Measurements were carried out using a microscope (DMLB, Leica) equipped with a camera (resolution = 2,600 × 2,600, Axiocam, Zeiss), and the KS300 3.0 system (Zeiss). A computer-based neuron tracing system (Neurolucida, Microbrightfield, Williston, Vermont) was used to trace single neurons. Spine density was assessed by counting the number of spines in five 20-μm dendrite segments per neuron chosen in second-to-fourth branch orders along apical and basal dendrites. All protrusions, with or without bulbous expansion, but no longer than 2 μm, were counted as spines if they were continuous with the dendritic shaft. The spine density (number of spines per 1-μm-long segment) were averaged for neuron. Total dendritic length was automatically calculated by the Neurolucida software after each neuron was traced. Spine the same dendritic segments as above by classifying individual spines as thin or large (stubby and mushroom) depending on their shape and dimension. Accordingly, spine head diameters were measured on previously acquired images using the ImageJ (NIH, USA) software. Spines were classified into two categories: “thin” (spine head diameter <0.55 μm) or “large” (=0.55 μm). All analyses were carried out by an experimenter blind to the experimental condition.

### Analysis of p38MAK and JNK phosphorylation

Quantification of phosphorylated protein kinases, [pTpY180/182]p38 MAPK or [pTpY183/185]JNK, was detected in protein extracts from EC slices, using two different ELISA kits (Millipore) as described in Origlia *et al*.^24^,^37^. Levels of phosphorylated forms were normalized with respect to the total p38 MAPK and total JNK protein content that was assessed using two ELISA kits purchased from the same company (Millipore). To verify whether p38MAPK and JNK activation have a neuronal localization we performed a double-immunostaining. Briefly, EC slices were fixed in 4% paraformaldehyde for 3 h immediately after the experiment, then cryoprotected in 30% sucrose PBS solution and finally cut at 25 μm thickness using a cryostate. Slices were washed three times in PBS, treated with a solution containing 0.5% Triton X-100, BSA 5% and horse serum 10%. Slices were then incubated with the following primary antibody:rabbit anti- (pThr180/pTyr182) p38MAPK polyclonal antibody (Lifespan, diluition 1:2000) and rabbit anti-(pThr 183 pTyr185) JNK polyclonal antibody (Chemicon; Diluition 1:100) overnightr at 4 °C; NeuN mouse anti-neuronal nuclei monoclonal antibody (Millipore; diluition 1:50) overnight at 4 °C. Then, sections were washed in 0.3% Triton X-100 in PBS and incubated in secondary antibodies (anti-mouse IgG and anti-rabbit IgG conjugated respectively with Alexa Fluor 488 and with Alexa Fluor 568, 1:500 dilution, Molecular Probes) diluted in 1% BSA in PBS for 2–3 h at room temperature, washed 3 × 10 min in PBS and cover slipped with Vectashield (Vector Laboratories). Serial optical sections were acquired using an Axio Imager. Z2 (Carl Zeiss) microscope and multi-channel images (transmitted fluorescences) were produced with ApoTome.2. High resolution images were obtained using EC Plan-NEOFLUAR 20×/0.5 objective. Control images of sections that were incubated without the primary antibody are reported in Supplemental Fig. S5.

### Statistical analysis

All data are reported as mean ± SEM. For electrophysiological experiments statistical comparisons between experimental groups or between FP amplitudes measured during baseline and after the induction protocol were performed by applying a two-way repeated-measures ANOVA with pair wise multiple comparison procedures (Holm–Sidak method, Sigmaplot 12.0). For behavioural experiments, a one-way ANOVA was applied to determine the differences in average discrimination indices and exploration rates in the test phase for each recognition task. One-sample t-tests were also used to determine whether the average discrimination index for each group was different from chance (hypothesized mean = 0). A one-way ANOVA was applied for evaluation of differences in spine density and activation of kinases between groups. Differences were considered significant when *p* < 0.05.

## Additional Information

**How to cite this article**: Criscuolo, C. *et al*. Entorhinal Cortex dysfunction can be rescued by inhibition of microglial RAGE in an Alzheimer's disease mouse model. *Sci. Rep.* **7**, 42370; doi: 10.1038/srep42370 (2017).

**Publisher's note:** Springer Nature remains neutral with regard to jurisdictional claims in published maps and institutional affiliations.

## Supplementary Material

Supplementary Figures
