# Supplementary material for: Entorhinal Cortex dysfunction can be rescued by inhibition of microglial RAGE in an Alzheimer’s disease mouse model
Source: Sci Rep. 2017 Feb 13;7:42370. doi: 10.1038/srep42370 (PMC5304222; doi:10.1038/srep42370)
Supplement: Supplementary Figures [file srep42370-s1.pdf]

Title: Entorhinal Cortex dysfunction can be rescued by inhibition of microglial RAGE in an Alzheimer's disease mouse model.

Authors: Chiara Criscuolo, Veronica Fontebasso, Silvia Middei, Martina Stazi, Martine Ammassari-Teule, Shirley Shi Du Yan, Nicola Origlia

Supplemental Figure 1

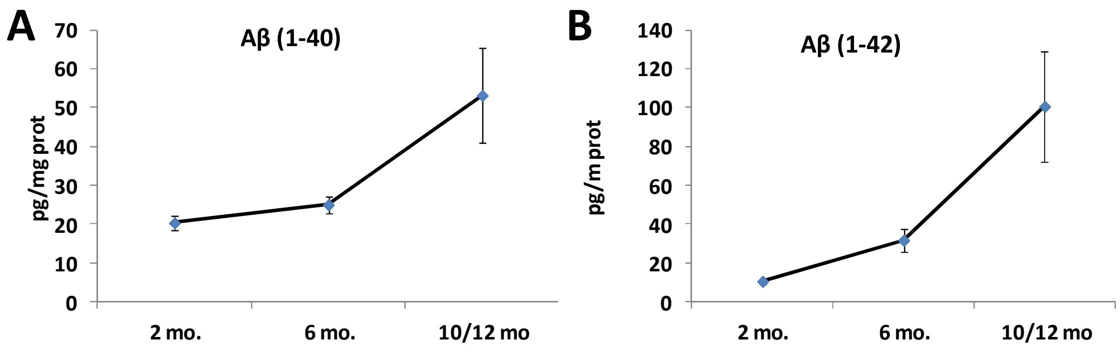

Supplemental Figure 1. Invitrogen human Aβ ELISA kits were used for the quantification of Aβ(1-42) and Aβ(1-40) in transgenic mice tissue homogenate following the procedure described in Origlia et al. (2014). A,B) Plots represent the average level of either Aβ (1-40)or Aβ(1-42) that were detectable in the range of pg/mg of total protein in EC slices from mhAPP mice of 2, 6 and 12 months of age (n= 6 slices each group). B) Aβ (1-42) levels were significantly higher in EC slices from 6 month old mhAPP than in 2 month old animals (10.5± 1.2 *vs* 31.7 ± 6.9 pg/mg, mice n = 3, slices n= 6, p<0.05).

Supplemental Figure 2

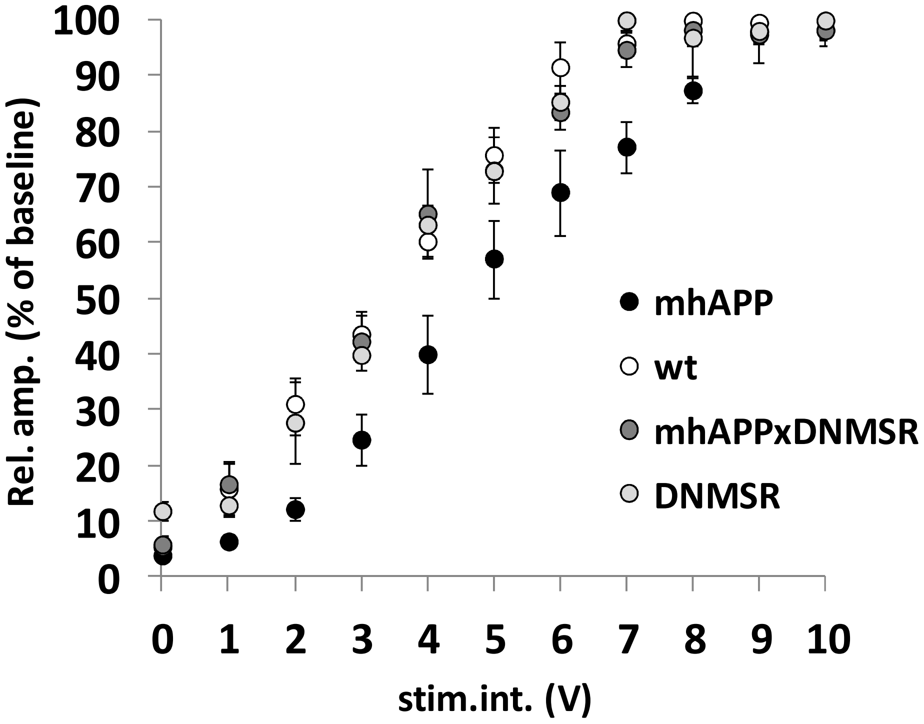

Supplemental Figure 2. Input–output curves in EC slices from 6 months old mice. The relative amplitude (Rel. Amp.) as a function of stimulus intensity (Stim.Int.) measured in volts (V) did not show significant differences between wild type controls (open circles) and DNMSR slices (light gray circles). In contrast, input/output relationship was altered in mhAPP mice (black circles). However synaptic transmission was normal in mhAPPxDNMSR slices (dark grey circles); the Input–output curve was significantly different from that recorded in mhAPP slices (at half maximal stimulation mean rel. Amp. were 73.2 ± 5.9 %, mice n = 3, slices n= 6, and 57.2 ± 7 % of baseline, mice n = 3, slices n= 6, respectively; $p<0.05$) and clearly overlapped with controls.

Supplemental Figure 3

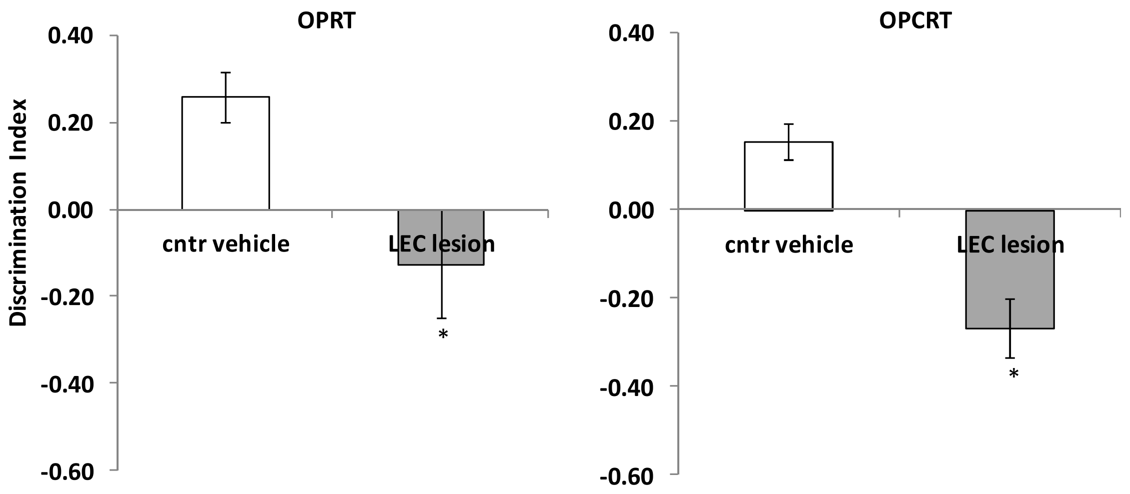

Supplemental Figure 3. Two months old C57BL/6J mice were anesthetized using a combination of zoletil and rampum (0.2 mg/kg), they were then placed in a stereotaxic frame. Following shaving the scalp, a midline incision was made and two holes were drilled bilaterally at stereotaxic coordinates targeting EC (1.8 mm posterior and 2.9 mm lateral from Bregma measured on the skull surface). An injecting needle was then inserted through the holes 2.4 mm below the dura and 0.3 µl of ibotenic acid (0.03 M ibotenate solution, Sigma Aldrich; dissolved in saline solution) was injected at slow injection rate to perform persistent lesions of lateral portion of the EC. Needle was left in situ 2 min after the end of injection, then scalp was sutured and mice back to their home cage for 7 days to recover from surgery before behavioural testing began. Sham operated controls underwent the identical procedure receiving only the vehicle solution (sterile phosphate buffer). Bars represent the average Discrimination indices that were calculated for OPRT (left) and OPCRT (right panel). Mice in the LEC lesion group showed no preference for exploring novel OP associations. Average discrimination indices were significantly different between vehicle and LEC lesion mice (0.15 ± 0.4, n = 3; *vs.* -0.27 ± 0.7; $p<0.05$). A similar results was obtained in lesion mice using the OPCRT. The average discrimination index was significantly different between LEC lesion group and vehicle injected control (-0.13 ± 0.1, n = 3; *vs.* 0.26 ± 0.6; $p<0.05$).

Supplemental Figure 4

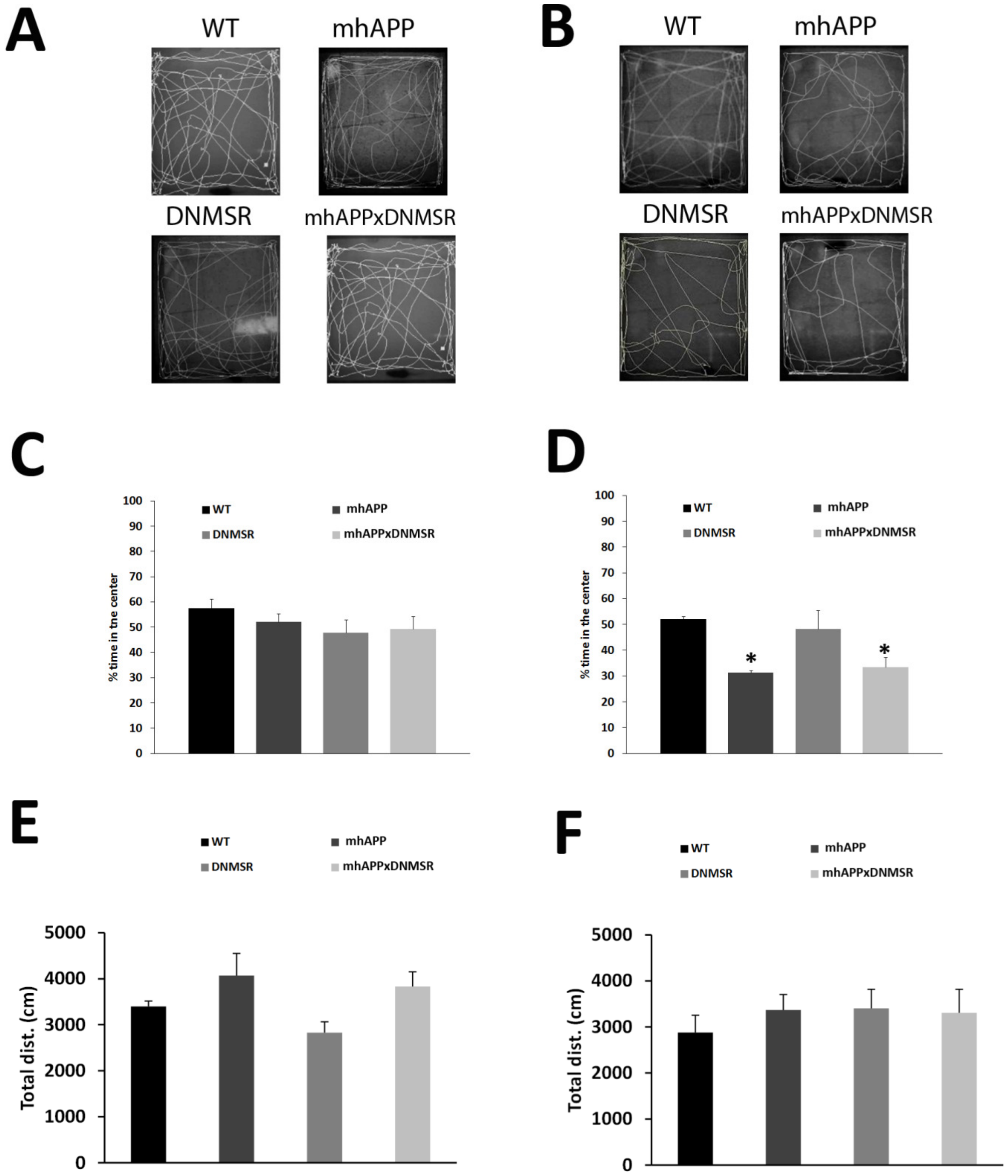

Supplemental Figure 4. During the habituation phase mice explored the box without object, for 5 min. In A, examples of traces obtained after mouse tracking during the exploration are reported for 2 month old animals of each group. In B, examples of traces obtained after mouse tracking during the exploration are reported for 6 month old animals of each group. C, No significant difference was found between group of 2 month old mice during exploration in the open field. In contrast, 6 month old mhAPP mice spent a

significantly lower amount of time in exploring the center of the arena, with respect to age-matched controls (31.25 ± 0.8 %, n = 6; *vs.* 52 ± 1 %; *p<0.05). A similar result was obtained in mhAPPxDNMSR mice (33. 5 ± 3.6 %, n = 6; *p<0.05 *vs.* WT and DNMSR; *p>0.05 *vs.* mhAPP). No significant differences were found in the total locomotor activity between either 2 month old (E) or 6 month old (F) groups of mice.

Supplemental Figure 5

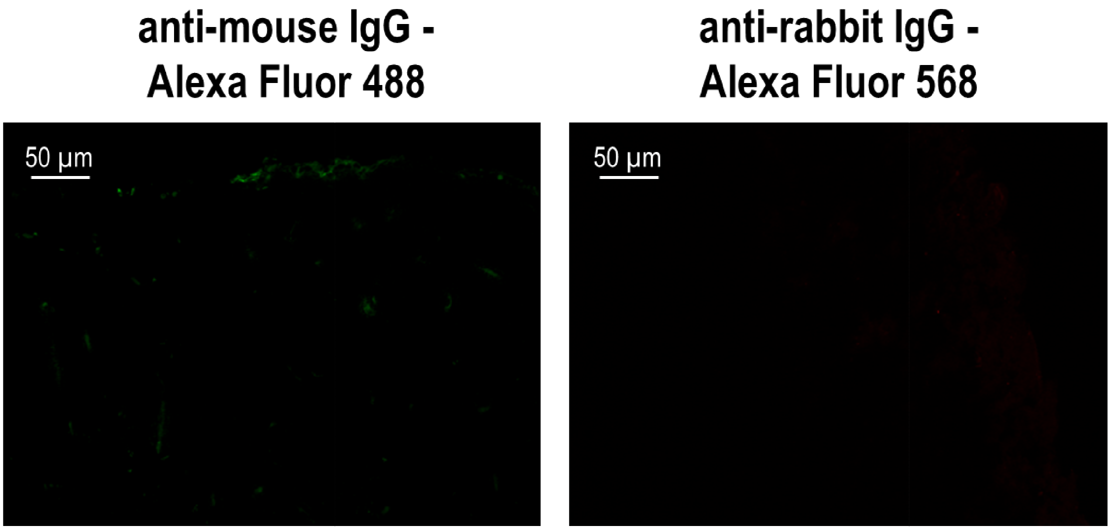

Figure S5. Immunohistochemistry in EC slices. Control experiments in sections that were incubated without the primary antibodies [anti-NeuN; anti-(p-p38) and anti p-JNK]. Representative images (20x) show the residual background staining due to the incubation with the secondary antibodies (anti-mouse IgG Alexa Fluor 488 conjugated in the left panel and anti-rabbit IgG Alexa Fluor 568 conjugated in the right panel.
